# Intraspecies plasmid and genomic variation of *Mycobacterium kubicae* revealed by the complete genome sequences of two clinical isolates

**DOI:** 10.1099/mgen.0.000497

**Published:** 2020-12-23

**Authors:** Jo Hendrix, L. Elaine Epperson, David Durbin, Jennifer R. Honda, Michael Strong

**Affiliations:** ^1^​ Computational Bioscience, University of Colorado Anschutz Medical Campus, Aurora, CO, USA; ^2^​ Center for Genes, Environment and Health, National Jewish Health, Denver, CO, USA; ^3^​ Advanced Diagnostics Laboratories, National Jewish Health, Denver, CO, USA

**Keywords:** hybrid genome assembly, MinION, whole-genome sequencing

## Abstract

*
Mycobacterium kubicae* is 1 of nearly 200 species of nontuberculous mycobacteria (NTM), environmental micro-organisms that in some situations can infect humans and cause severe lung, skin and soft tissue infections. Although numerous studies have investigated the genetic variation among prevalent clinical NTM species, including *Mycobacterium abscessus* and *Mycobacterium avium *, many of the less common but clinically relevant NTM species, including *M. kubicae *, still lack complete genomes to serve as a comparative reference. Well-characterized representative genomes for each NTM species are important both for investigating the pathogenic potential of NTM, as well as for use in diagnostic methods, even for species that less frequently cause human disease. Here, we report the complete genomes of two *M. kubicae* strains, isolated from two unrelated patients. Hybrid short-read and long-read sequencing and assembly, using sequence reads from Illumina and Oxford Nanopore Technologies platforms, were utilized to resolve the chromosome and plasmid sequences of each isolate. The genome of NJH_MKUB1 had 5135 coding sequences (CDSs), a circular chromosome of length 5.3 Mb and two plasmids. The genome of NJH_MKUB2 had 5957 CDSs, a circular chromosome of 6.0 Mb and five plasmids. We compared our completed genomic assemblies to four recently released draft genomes of *M. kubicae* in order to better understand intraspecies genomic conservation and variability. We also identified genes implicated in drug resistance, virulence and persistence in the *M. kubicae* chromosome and plasmids. Virulence factors encoded in the genome and in the plasmids of *M. kubicae* provide a foundation for investigating how opportunistic environmental NTM may cause disease.

## Data Summary

Data generated in this study were deposited in GenBank as BioSamples SAMN12910285, SAMN12910286 and SAMN16828374, under BioProject ID PRJNA575747. Additional comparative data were accessed from GenBank assembly accession numbers GCA_002101745.1, GCA_001667525.1, GCA_001673455.1 and GCA_010723135.1.

**Impact Statement**

This study describes the first complete reference genomes of *Mycobacterium kubicae *. NJH_MKUB1 and NJH_MKUB2 were sequenced using Illumina and Oxford Nanopore Technologies and assembled to high-quality completion using hybrid methods, enabling a more thorough understanding of the pan genome of *M. kubicae *, including virulence factor identification, and providing resolution to distinguish between genes integrated on the chromosome and mobile elements located on plasmids. The high variability in genome size, content and plasmid abundance was apparent by examining members of the same bacterial species, and within the two samples we sequenced. NJH_MKUB1 had a chromosome of length 5.3 Mb and two plasmids, while NJH_MKUB2 had a chromosome of length 6.0 Mb and five plasmids. Chromosome lengths and numbers of plasmids varied dramatically between strains, but *M. kubicae* shared a strong set of core genes involved in metabolism and other cellular processes. We also identified a number of potential drug-resistance and virulence factors throughout the genome of this opportunistic pathogen. The findings presented here contribute to our understanding of the coding capacity, genomic plasticity and plasmid variability of nontuberculous mycobacteria, and provide two robust reference genomes of *M. kubicae* for subsequent comparative studies.

## Introduction

Mycobacteria comprise a genus of nearly 200 species that range from obligate human pathogens, such as *Mycobacterium tuberculosis* and *Mycobacterium leprae *, to the environmental nontuberculous mycobacteria (NTM) species. NTM occupy a wide array of environmental niches, particularly thriving in soil and water biofilms from natural and built environments [1]. As opportunistic environmental pathogens, NTM can cause disease in immunocompromised patients, as well as individuals with no apparent pre-existing susceptibility [1–3]. *Mycobacterium kubicae* is a relatively rare NTM that is clinically significant as a cause of pulmonary disease [4, 5]. Previous studies have shown that it is resistant to amikacin and rifampicin, and partially resistant to the first-line anti-tuberculosis drugs isoniazid and ethambutol [4]. Four *M. kubicae* genome assemblies (CIP 106428, ACS1160, 1146974.2 and JCM 13573) were published as of August 2020, but none were complete, non-gapped assemblies.

Complete genome assemblies enhance our understanding of micro-organisms by revealing the full protein coding capacity of the entire chromosome and plasmids, and by identifying repeat regions and integrated phages. Incomplete assemblies, in contrast, are typically fragmented into contigs, which may split gene clusters and disrupt ORFs, impeding operon analysis and comparison of gene profiles. Often appearing as short segments of DNA in incomplete genomes, plasmids may be disrupted or even mistaken for unattached chromosomal segments. As opposed to *M. tuberculosis *, which is thought not to naturally retain plasmid elements [6], NTM are known to harbour plasmids of varying sizes, numbers, composition and coding capacity [7]. NTM plasmids enhance cellular survival [8], pathogenesis [9] and antimicrobial resistance [10], yet remain understudied. As a result, we lack understanding of how these mobile elements might influence the pathogenic potential of various NTM species.

Until recently, plasmids were difficult to identify from short-read whole-genome sequence data alone. Illumina next generation reads are highly accurate and are used in most microbial genome re-sequencing projects. However, Illumina reads have relatively short read lengths with a maximum of 250–300 bp depending on the chemistry and instrument used. These short reads can often lead to incomplete assemblies with more than desirable fragmentation in high G+C and highly repetitive regions. Mycobacterial genomes have high G+C content (63–67 mol%) and high occurrences of insertion sequences (ISs), which often have inverted repeats on the ends; these factors present problems for assembly of mycobacterial genomes using short-read sequencing alone [11]. The much longer sequencing reads produced by third-generation technologies like Oxford Nanopore Technologies (ONT) [12] and PacBio span larger regions of the genome, resulting in more complete assemblies; however, long-read sequencing remains less accurate in basecalling per base than Illumina short-read sequencing.

In this study, we present the first two completed genome assemblies of *M. kubicae *. The two strains, called here NJH_MKUB1 and NJH_MKUB2, were recovered from bronchoalveolar lavage samples of two patients. We sequenced the clinical isolates using Illumina paired-end and ONT long-read technologies, then used a hybrid assembly pipeline [13] to resolve the chromosome and plasmids of each strain. Genes related to drug resistance were identified on the chromosomes and in the plasmids. Through genomic comparisons, a core set of genes common across the *M. kubicae* genomes were identified. A high degree of variability in the plasmid content and occurrence between strains was also observed, suggesting that *M. kubicae* can add and remove genetic content through plasmids and chromosomal content. These findings highlight the need for complete, non-gapped genomic assemblies of both common and rare NTM, and reveal the benefits of whole-genome intraspecies isolate comparisons.

## Methods

### Library preparation and sequencing

Two patients’ bronchoalveolar lavage isolates were sent to the National Jewish Health Advanced Diagnostics Mycobacterial Laboratory for NTM species identification. NJH_MKUB1 was recovered from a 63-year-old woman and NJH_MKUB2 was recovered from an 86-year-old man. Both were identified as *M. kubicae* using *rpoB* locus Sanger sequencing. After species identification, culture slants were provided for scaled up microbiological culture in order to obtain sufficient high molecular mass DNA for downstream sequencing. From slants, isolates were streaked for purity onto Middlebrook 7H10 agar plates and incubated at 37 °C for 21 days. After culture, a typical yellow, scotochromogenic pigment was noted. From plates, one colony per isolate was picked and aseptically inoculated into 50 ml Middlebrook 7H9 culture media, incubated for 21 days, and expanded into a 250 ml 7H9 culture to generate a large quantity of cells for genomic DNA (gDNA) extraction and long-read sequencing on the ONT MinION platform. gDNA was extracted using the method described by Epperson and Strong [14] for paired-end and long-read sequencing.

### Illumina sequencing

Isolates were sequenced using Illumina 2×250 chemistry with a Nextera DNA Flex library preparation to depths of 80.7× and 59.9×. Illumina paired reads were trimmed by skewer v0.2.2 [15] using an adapter sequence of 5′-CTGTCTCTTATACACATCT-3′, a minimum length of 40 bases after trimming and end quality of 20. Read quality was assessed using fastQC v0.11.8.

### ONT MinION sequencing

One microgram of DNA was packaged into a library for ONT sequencing. DNA libraries were prepared using the ligation sequencing kit (SQK-LSK109; ONT) with the native barcoding kit (EXP-NBD104; ONT), according to the manufacturer’s instructions. MinION sequencing proceeded for 21 h on a R9.4.1 flow cell. NJH_MKUB1 was basecalled and demultiplexed with Guppy v3.0.3. NJH_MKUB2 was basecalled and demultiplexed with Guppy v3.2.2 [13]. Additional demultiplexing was performed using Porechop v0.2.4 (https://github.com/rrwick/Porechop) and only reads sorted into the same barcode bin by both software packages were used in downstream analysis. ONT reads were filtered for a minimum length of 5000 bp and a minimum mean quality score of 20. After filtering, the calculated long-read coverage of NJH_MKUB1 and NJH_MKUB2 was 232.7× and 367.5×, respectively.

### Genome assembly

Four additional *M. kubicae* datasets from the National Center for Biotechnology Information (NCBI) were accessed: CIP 106428, ACS1160, 1146974.2 and JCM 13573. Each assembly was downloaded in fasta format for further analysis. CIP 106428 was sequenced with Illumina HiSeq to 100× coverage then assembled with Spades v3.5.0 [16] into 116 contigs (GenBank accession no. GCA_002101745.1) [7]. ACS1160 was sequenced with Illumina NextSeq 500 to 355.6× coverage then assembled with Spades v3.1.1 into 54 contigs (GenBank accession no. GCA_001667525.1). 1146974.2 was sequenced with Illumina NextSeq 500 to 293.2× coverage then assembled with Spades v3.1.1 into 244 contigs (GenBank accession no. GCA_001673455.1). JCM 13573 was sequenced with Illumina HiSeq 2500 to 196× coverage and with ONT MinION then assembled with Canu v1.7 into 5 contigs of lengths 2.9 Mb, 1.8 Mb, 888 kb, 388 kb and 42.3 kb (GenBank accession no. GCA_010723135.1) [17]. We downloaded and reassembled the raw JCM 13573 short and long reads using Unicycler v0.4.4 [18].

The genomes for NJH_MKUB1 and NJH_MKUB2 were assembled using the short-read, long-read and hybrid assembly methods offered by Unicycler v0.4.4 [18]. The long reads of NJH_MKUB2 were also assembled using Canu v2.0 [19]. Assembly accuracy was estimated by the number of variations between the Illumina reads and assembled sequences as detected by Bowtie2 [20]. Completed chromosomes were reoriented to start at the *dnaA* gene [18].

### Gap repair

After hybrid assembly, there was still a gap in the NJH_MKUB2 chromosome that prevented closure. The flanking regions were aligned to the short-read, long-read Unicycler and long-read Canu assemblies. The Canu assembly was selected for the reference in this region because the Illumina contigs did not cover the gap and the long-read Unicycler assembly had lower accuracy (see Results).

To resolve the gap, the 4996 upstream bases were replaced with a 137 bp sequence, the ends were connected and the chromosome was reoriented to the *dnaA* gene. Long reads were aligned to the completed assembly using Bowtie2 and the number of variants (>20 % of reads had non-reference allele) within 2000 bp of the connection site were counted. In brief, the ends of the gapped sequence were directly connected, and the chromosome was reoriented so the long reads would have room to align. Four reads aligned to the connection point with 554 variants. In the resolved sequence, 16 reads spanned the connected point with only two variants. Thus, the resolved sequence is used for the remainder of this text. The resolved region of NJH_MKUB2 is located at positions 5 312 858–5 312 994.

The 4 996 bp deleted sequence aligned with 99 % similarity to the plasmid, pMKUB2_3 at positions 25 257–30 242, so this segment was not added as an independent contig. Half of the deleted sequence also had 77 % identity to the region directly upstream of its original position in the chromosome. We propose that the short reads could not differentiate between the similar sequences of pMKUB2_3 and the chromosome, so the more abundant plasmid sequence was incorrectly added to the chromosome. This was not fixed during hybrid assembly because Unicycler uses the short reads to (incorrectly in this case) error-correct the long reads. The long-read-only assembly was needed to resolve the repetitive region to close the gap.

### Annotation and genomic analysis

**Fig. 1.**
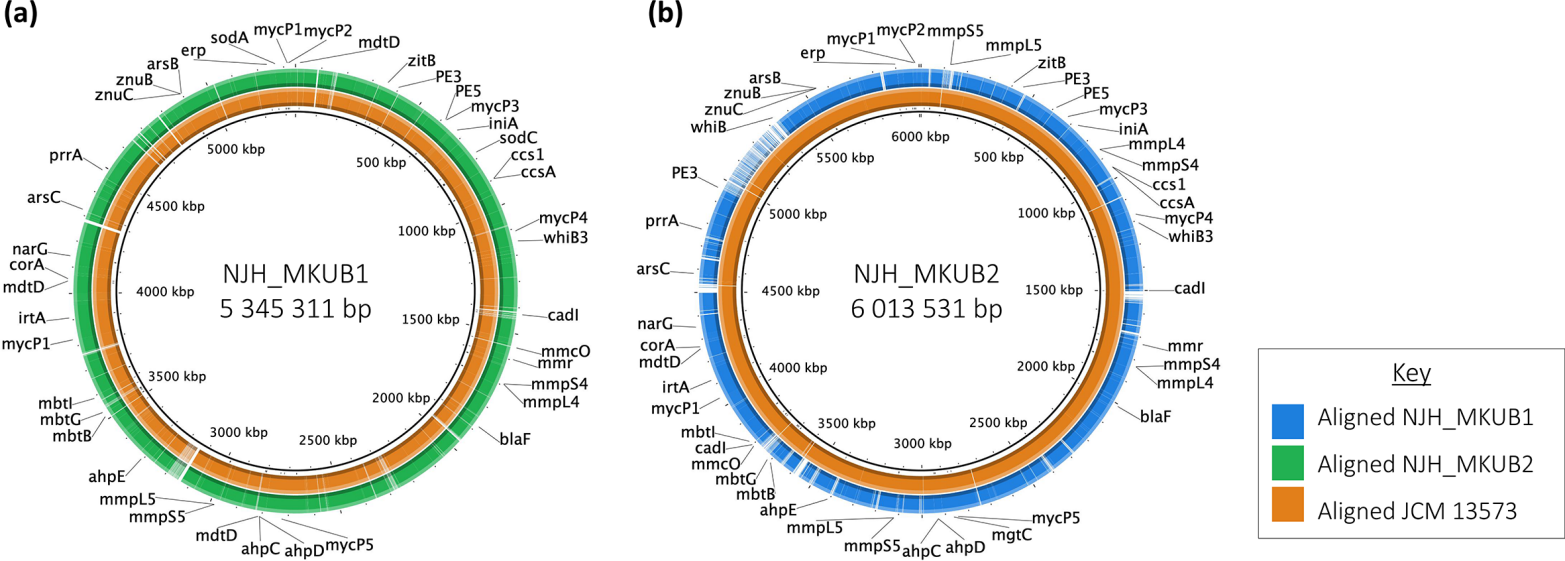
Genome maps and alignment of NJH_MKUB1 and NJH_MKUB2. Genome maps showing the chromosomes of strain NJH_MKUB1 (a) and NJH_MKUB2 (b). Rings from inner to outer layer represent the location of CDSs, alignment to the chromosome sequences of the other strain, and genes of interest.

Hybrid assemblies were annotated with Prokka v1.13.3 [21] to identify potential coding sequences (CDSs), tRNAs, ISs [11] and rRNA. The previously published assemblies were re-annotated with Prokka for comparative analysis. The identified 16S rRNAs were aligned against the consensus sequence for the original *M. kubicae* species identification generated by Floyd *et al*. [4] in order to confirm the species. Phylogenetic relatedness was determined through average nucleotide identities (ANIs) of the full genomes using OrthoANI [22] and CDS profiles using Roary [23]. The pan and core genome were computed using Roary and displayed using Phandango [24]. Functional annotation groups were assigned to the core gene set using GhostKOALA [25], then grouped for ease of interpretation. Functional annotations of specific CDSs in NJH_MKUB1 and NJH_MKUB2 were assigned by Prokka and GhostKOALA, then confirmed through the UniProt [26] database or another listed source. The JCM 13573 reassembly was not used for chromosomal comparison, because it was structurally similar to NJH_MKUB2 (Fig. 1b).

### Plasmid detection

To detect potential plasmids, contigs <1 Mb in length from the hybrid assemblies of NJH_MKUB1, NJH_MKUB2 and the JCM 13573 reassembly were tested for homology against known plasmids and checked for circularity. Contigs that met one of these criteria were considered potential plasmids. Homology of contigs to existing plasmids was detected using Microbial Nucleotide blast of the NCBI Complete Plasmid database (accessed November 8 2020). A hit was retained if the aligned segment covered at least 75 % of the contig. Contigs were analysed for circularity using Unicycler. During assembly, Unicycler checked contig ends for significant overlap, which would indicate that the contig was circular [18]. brig [27] was used to generate circular alignment plots of plasmids to similar sequences.

## Results

### Illumina sequencing and assembly

Illumina HiSeq sequencing produced 1 744 896 and 1 435 914 reads for NJH_MKUB1 and NJH_MKUB2, respectively. The assemblies generated were fragmented into 99 contigs for NJH_MKUB1 and 245 contigs for NJH_MKUB2, consistent with the previously published Illumina-only assemblies of *M. kubicae* that were resolved into 54 to 244 contigs each (Table 1).

**Table 1.** Assembly statistics for *M. kubicae* isolates used in this study

Strain	Technology	Assembly length (bp)	No. of contigs	Coverage
NJH_MKUB1	Hybrid	5 463 379	3	312.56×
NJH_MKUB2	Hybrid	6 267 376	6	424.74×
JCM 13573	Hybrid	6 016 837	3	196.00×
CIP 106428	Illumina	5 832 568	116	100.00×
ACS1160	Illumina	5 618 076	54	355.63×
1146974.2	Illumina	5 534 339	244	293.21×

### ONT sequencing and assembly

ONT long-read sequencing on the MinION yielded 153 114 reads, encompassing 1 271 389 991 bases, for NJH_MKUB1, and 292 598 reads, encompassing 2 305 166 903 bases, for NJH_MKUB2, after demultiplexing and filtering. Using only ONT reads, the NJH_MKUB1 genome was assembled into three contigs; however, there were 42 913 variations between the ONT assembly and Illumina reads. ONT reads for the NJH_MKUB2 genome were assembled by Unicycler into 15 contigs with 64 332 variations from the Illumina reads. Canu assembled the NJH_MKUB2 ONT reads into 27 contigs with 4560 variations.

### Hybrid assembly of Illumina and ONT reads

Using hybrid assembly, the NJH_MKUB1 genome was resolved into three contigs and the NJH_MKUB2 genome was resolved into six contigs (Table 1). In addition to completeness, accuracy also improved. The number of variations between the hybrid assemblies and the respective Illumina read sets decreased to 89 in NJH_MKUB1 and 112 in NJH_MKUB2, indicating that there were fewer errors in the sequence (0.2 and 0.17 %, respectively). Due to the improved completeness and accuracy of the hybrid assemblies compared to the Illumina-only or ONT-only methods, only the hybrid assemblies for NJH_MKUB1 and NJH_MKUB2 were used for analysis.

The NJH_MKUB1 genome was 5 463 379 bases in length with 66.3 mol% G+C content, 5135 CDSs and 53 tRNAs. The genome consisted of three elements: a circular chromosome of length 5 345 311 bp and two plasmids. The NJH_MKUB2 genome was 6 267 376 bases in length with 66.0 mol% G+C content, 5957 CDSs and 52 tRNAs. The genome consisted of six elements: a circular chromosome of length 6 008 535 bp and five plasmids (Table 1).

JCM 13573 was reassembled into three contigs. The entire genome was 6 016 837 bp in length with 66.0 mol% G+C content, 5576 CDSs and 51 tRNAs. The published assembly had 264 variants, while the reassembly had 172 variants compared to the Illumina reads. The genome consisted of three elements: a circular chromosome of length 5 958 133 bp and two closed plasmids (Table 1).

### *M. kubicae* description

The 16S rRNA sequences of NJH_MKUB1, NJH_MKUB2, CIP 106428, 114674 and JCM 13573 each possessed 100 % sequence identity to the Floyd *et al*. consensus sequence [4], suggesting these isolates are *M. kubicae *. ACS1160 contained a T to C polymorphism at position 206. An alignment search of the ACS1160 16S sequence against published 16S sequences on blast did not yield a better match to any other species; thus, this sample was retained in our analysis.

**Fig. 2.**
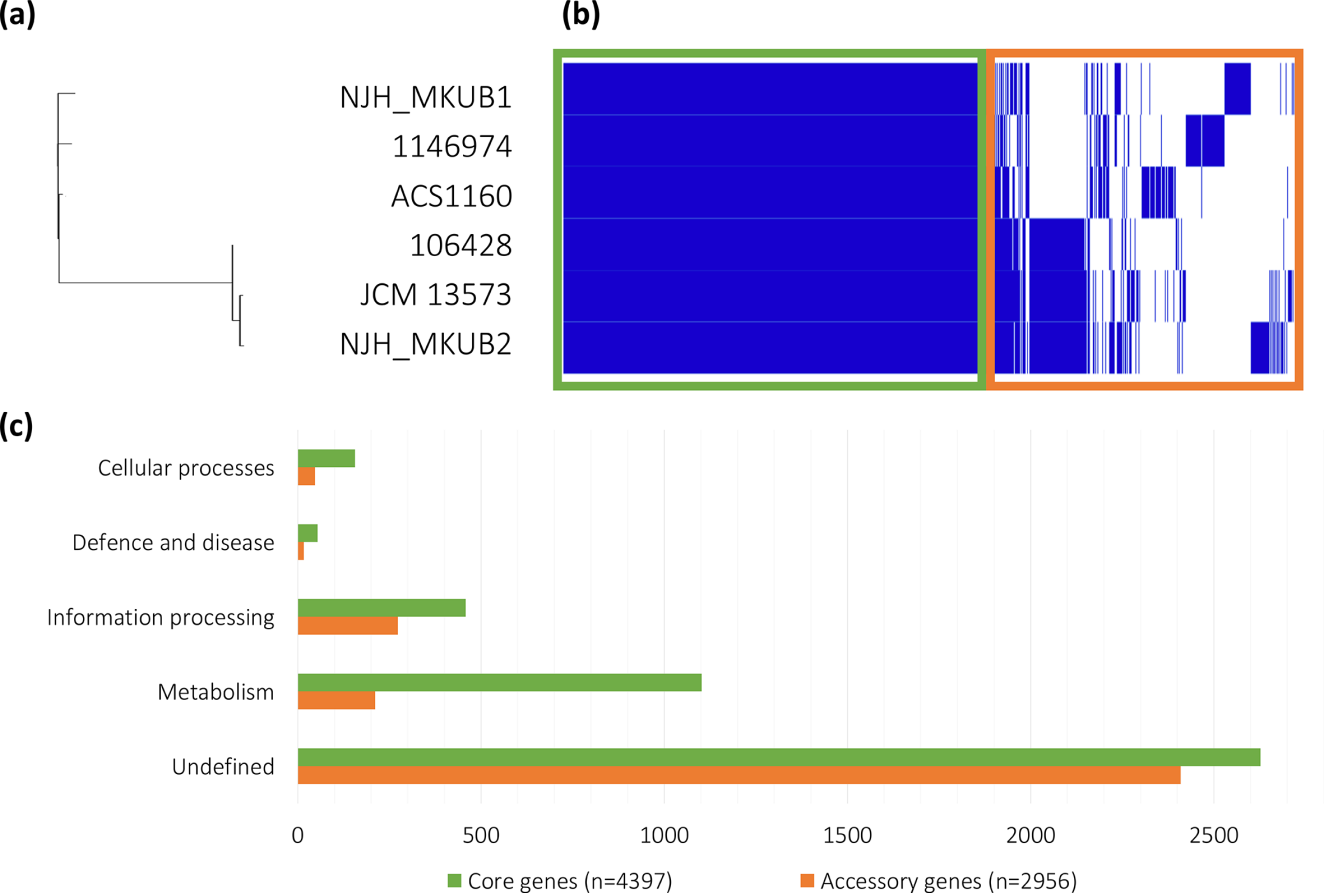
Relatedness and pan genome of *M. kubicae* strains. (a) Phylogenetic relationship between the six *M. kubicae* strains analysed in this study, based on CDS comparison. (b) The pan genome consists of 7353 genes in total, depicted across the *x*-axis. The presence of a gene in each strain is represented with a blue tick. Core genes (4397) found in all strains are boxed in green. Accessory genes (2956) missing in at least one strain are boxed in orange. (c) Number of core (green) and accessory (orange) genes assigned to each broad functional annotation group.

Through CDS analysis, NJH_MKUB1 was found to be most closely related to ACS1160, while NJH_MKUB2 was most closely related to JCM 13573 and CIP 106428 (Fig. 2a). These relationships were confirmed through ANI, where NJH_MKUB1 had 98.22 % ANI to ACS1160 and NJH_MKUB2 had 99.96 % ANI to JCM 13573.

### Pan-genome analysis of all available *M. kubicae* genome assemblies

The pan genome, consisting of core (present in >95 % of strains) and accessory genes, contained 7353 genes (Fig. 2b). Of the 4397 core genes, 1770 (40.3 %) were assigned to functional annotation groups. The core contained 156 genes involved in cellular processes, 54 genes involved in xenobiotic defence (*n*=46) and human disease (*n*=8), and 458 genes involved in information processing. A majority of the core genes were involved in metabolism (*n*=1102), including the metabolism of carbohydrates (*n*=239), amino acids (*n*=127), cofactors and vitamins (*n*=119), energy (*n*=112), lipids (*n*=93), nucleotides (*n*=69), glycan biosynthesis (*n*=44), terpenoids and polyketides (*n*=29), secondary metabolites (*n*=2), and otherwise unclassified (*n*=174) or grouped protein families (*n*=75) (Fig. 2c).

There were 2956 accessory genes found in at least one, but not all, of the strains (Fig. 2b). Only 547 (18.5 %) of the accessory genes could be assigned a functional group (Fig. 2c). A much smaller proportion of the accessory genes were involved in metabolism, which is consistent with metabolism’s role as a central core function.

### Chromosomal analysis

Chromosome analysis was performed on NJH_MKUB1 and NJH_MKUB2, where the quality of the assemblies allowed the complete chromosomes to be extracted for individual analysis. The chromosomes contained a number of genes linked to tolerating and sequestering various metals. Additionally, the chromosomes contained genes encoding the MmpL4/MmpS4 and MmpL5/MmpS5 proteins, PE/PPE proteins, and parts of the ESX type VII system including a complete copy of ESX-1. The strains structurally differed at position 4 680 314 in NJH_MKUB1 and 4 958 296 in NJH_MKUB2, where NJH_MKUB2 had a 386 309 bp insert that included a PE3 gene and a partial ESX-5 system (Fig. 1a, b).

### Plasmid characterization

Nine contigs were investigated as potential plasmids: two from NJH_MKUB1, five from NJH_MKUB2 and two from the JCM 13573 reassembly. Each plasmid had either significant sequence similarity to an existing plasmid (*n*=5) and/or were circularized during assembly (*n*=7). Plasmids pMKUB1_1 in NJH_MKUB1 and pMKUB2_1 in NJH_MKUB2 were not circularized during assembly because neither contig had significant overlap of the ends. Evidence supporting contigs as separate pieces of the same plasmid element were not found. Thus, all potential plasmids from NJH_MKUB1, NJH_MKUB2 and JCM 13573 were determined to be individual plasmids (Table 2).

**Table 2.** Plasmids identified in *M. kubicae
*

Strain	Plasmid name	Accession no.	Length (bp)	No. of CDSs	Status
NJH_MKUB1	pMKUB1_1	CP045082	96 951	112	Incomplete
NJH_MKUB1	pMKUB1_2	CP045083	21 117	22	Circular
NJH_MKUB2	pMKUB2_1	CP045076	108 396	121	Incomplete
NJH_MKUB2	pMKUB2_2	CP045077	68 667	80	Circular
NJH_MKUB2	pMKUB2_3	CP045078	34 639	37	Circular
NJH_MKUB2	pMKUB2_4	CP045079	26 209	33	Circular
NJH_MKUB2	pMKUB2_5	CP045080	20 930	23	Circular
JCM 13573	pJCM_13573_1	CP065048	30 582	30	Circular
JCM 13573	pJCM_13573_2	CP065049	28 122	28	Circular

**Fig. 3.**
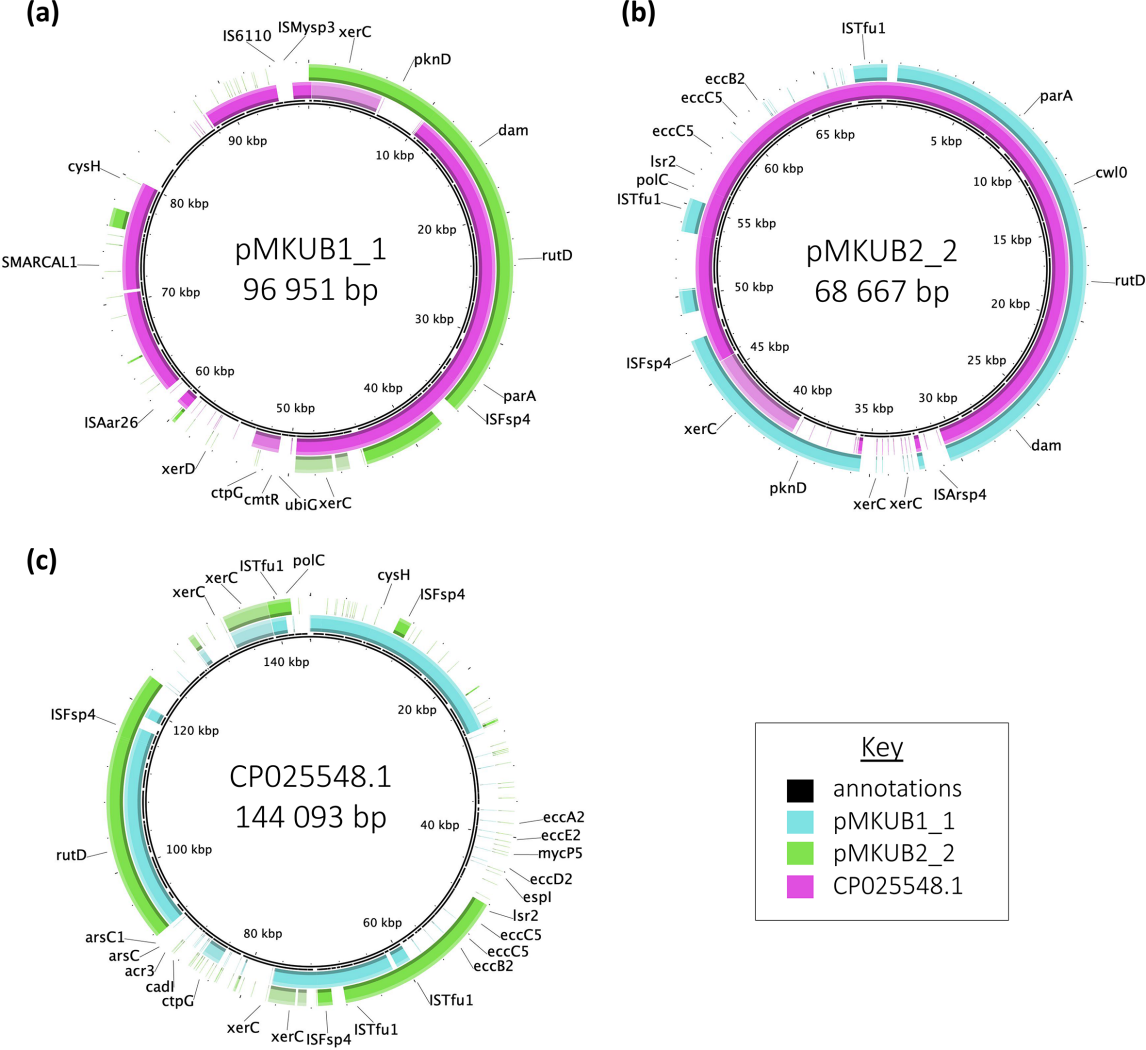
Comparison of plasmids pMKUB1_1, pMKUB2_2 and CP022548.1. Gene maps of pMKUB1_1 (a), pMKUB2_2 (b) and CP025548.1 (c). The rings from inner to outer layer represent the location of CDS annotations, sequence alignment to the other two plasmids and annotated CDSs.

Plasmid pMKUB1_1 was 96 951 bp with 111 potential CDSs and had 76 % coverage and 99.89 % sequence identity to *Mycobacterium paragordonae* unnamed plasmid 2 (accession no. CP022548.1) (Fig. 3a). Plasmid pMKUB2_2 was 68 667 bp with 80 CDSs and had 86 % coverage and 99.93 % sequence identity to plasmid CP022548.1 (Fig. 3b). Plasmid CP022548.1 was much larger at 144 093 bp with 154 CDSs. Though pMKUB1_1 and pMKUB2_2 had 99.93 % sequence identity to each other and a high identity to CP022548.1, they only overlapped at a portion of their positions, approximately 45 kb (Fig. 3c). Only pMKUB2_2 and CP022548.1 shared regions that included virulence gene controller (*lsr2*) and members of the ESX system (*eccC5*, *eccB2*). CP022548.1 had additional ESX-2 genes along with an arsenic-tolerance gene (*arsC*) and a cadmium-tolerance gene (*cadI*). Though not present in any of the plasmids, the genes specific to CP022548.1 were all present on the chromosomes of NJH_MKUB1 and NJH_MKUB2 (Fig. 1). The three plasmids likely diverged from an ancestral plasmid and all included multiple ISs, but only two were shared across all three plasmids (IS*Fsp4*, IS*Tfu1*).

**Fig. 4.**
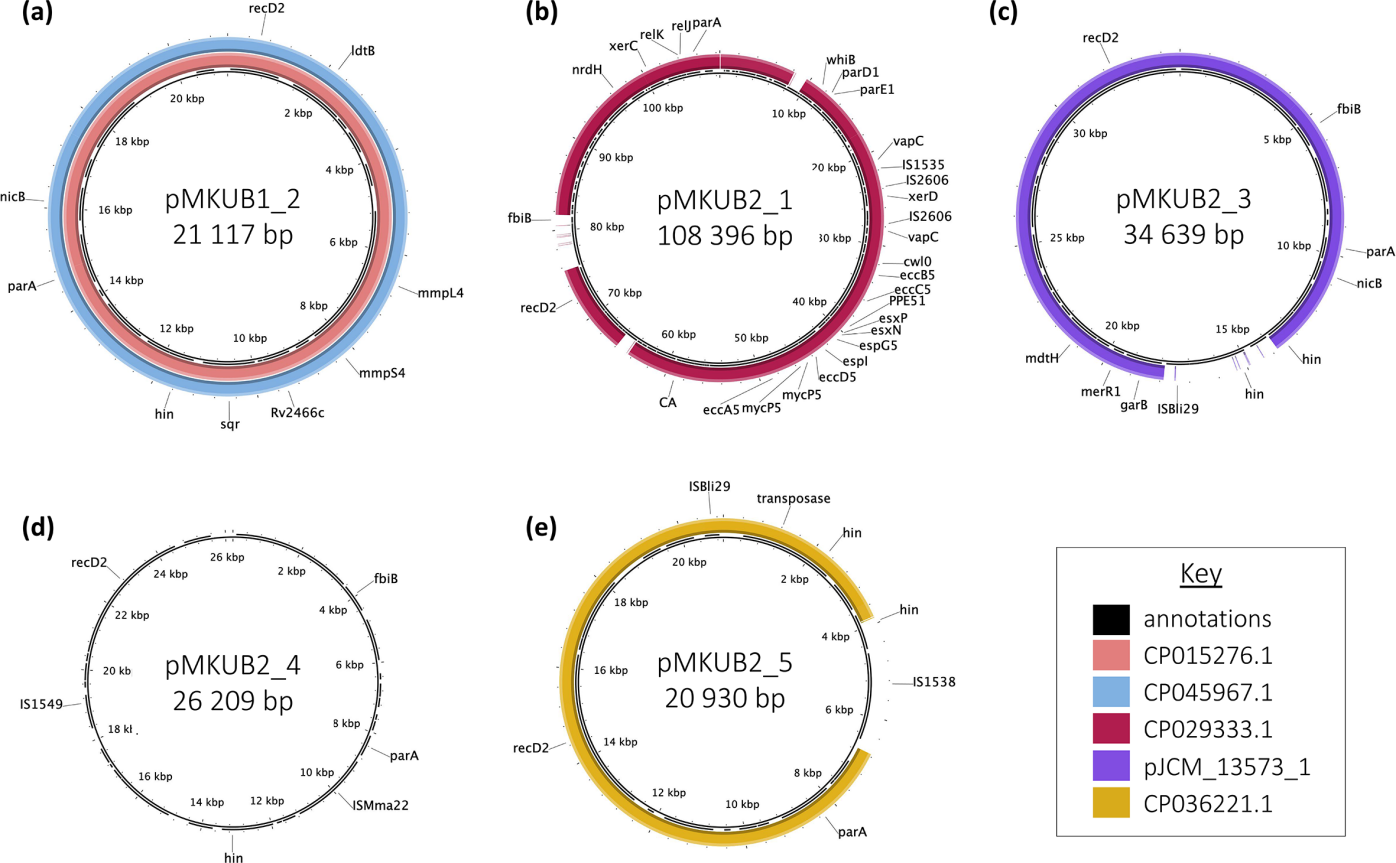
Genome maps of *M. kubicae* plasmids pMKUB1_2, pMKUB2_1 and pMKUB2_3–5. The rings from inner to outer layers represent the location of CDSs, sequence alignment and genes of interest. (a) pMKUB1_2 aligned to plasmids CP015276.1 and CP045967.1. (b) pMKUB2_1 aligned to plasmid CP029333.1. (c) pMKUB2_3 aligned to pJCM_13573_1. (d) pMKUB2_4 did not have significant sequence similarity to any sequences. (e) pMKUB2_5 aligned to plasmid CP036221.1.

Plasmid pMKUB1_2 was 21 117 bp with 22 CDSs and was similar to previously reported *Mycobacterium chimaera* plasmids with accession numbers CP045967 [28] and CP015276 [29]. The plasmid included a β-lactam target (*ldtB*), transmembrane proteins (*mmpL4/mmpS4*), nicotine degradation gene (*nicB*) and TP053 resistance mutation (Rv24662) (Fig. 4a).

Plasmid pMKUB2_1 was 108 396 bp with 120 CDSs and showed high sequence identity to pMAC109a (GenBank accession no. CP029333) [30]. Both plasmids contained a toxin–antitoxin system (*relK*, *relJ*) and a cluster of ESX-5 genes. Compared to pMAC109a, this plasmid contained an insertion for a protein involved in the biosynthesis of the pathway coenzyme F420 (*fbiB*) (Fig. 4b).

Plasmid pMKUB2_3 was 34 639 bp with 37 CDSs and included a multidrug-resistance gene (*mdtH*), mercury-tolerance gene (*merR1*) and nicotine-degradation gene (*nicB*). This plasmid did not have high sequence identity or coverage with previously reported sequences. Plasmid pJCM_13573_1 was 30 582 bp with 30 CDSs and similar to pMKUB2_3, but had a 4000 bp deletion (Fig. 4c).

Plasmid pMKUB2_4 was 26 209 bp with two IS elements (IS*Mma22* and IS*1549*) and 33 CDSs, including a protein involved in biosynthesis of the pathway coenzyme (*fbiB*). This plasmid did not have high sequence identity or coverage with previously reported sequences (Fig. 4d).

Plasmid pMKUB2_5 was 20 930 bp with 23 CDSs and similar to unnamed plasmid one from '* Mycobacterium avium* subsp. *hominissuis *' strain mc2 2500 (accession no. CP036221). The plasmid consisted predominantly of transposases and invertases (Fig. 4e).

Plasmid pJCM_13573_2 was 28 122 bp with *mmpL4/mmpS4* and *mmpL5/mmpS5* genes. This plasmid did not have high sequence identity or coverage with previously reported sequences (results not shown).

Seven plasmids were identified in NJH_MKUB1 and NJH_MKUB2, and though some plasmids contained the same genes or regions, no entire plasmid was present in both, suggesting that plasmids were acquired independently, likely in response to specific environmental needs rather than being maintained across the entire *M. kubicae* species.

## Discussion

### Sequencing and assembly

The hybrid assembly of Illumina and ONT reads generated superior genome assemblies, more complete than the Illumina-only assemblies and more accurate than the ONT-only assemblies. The improvement in accuracy is attributed to the read polishing process, where the more accurate Illumina reads were used to correct discrepancies in the long reads during assembly [18]. Variations in the form of SNPs and small indels were still detected between the hybrid assemblies and the respective Illumina read sets as can be expected, since errors [31] and coverage gaps can occur in Illumina sequencing.

NJH_MKUB1 had a full genome size of 5 463 379 bp, which included a circular chromosome of length 5 345 311 bp and two plasmids. NJH_MKUB2 was considerably larger, having a full genome size of 6 267 376 bp with a chromosome of length 6 008 535 bp and five plasmids. The extra content in NJH_MKUB2 appears in small segments throughout the chromosome and a larger insert between positions 4.9 and 5.5 Mb (Fig. 1).

Four draft assemblies of *M. kubicae* were previously published, but none were complete with circularized chromosomes. JCM 13573 was the most complete genome to date, having resolved into five contigs where the largest contig was about half the size of the two chromosomes characterized in the present study. We reassembled the JCM 13573 data using the most current tools, and this effort resulted in a complete assembly with one closed chromosome and two circularized plasmids.

### Pan-genome analysis

Pan-genome analysis of the six available strain assemblies revealed a set of 4397 core genes involved in cellular processes, cell defence and human disease, information processing, and metabolism. A majority of the core genes were involved in metabolism, consistent with the critical role of metabolism as a core function.

All strains had the gene *iniA*, which has been implicated in improved tolerance to isoniazid and ethambutol. This is consistent with the findings of Floyd *et al*., who reported that clinical isolates were partially resistant to these two drugs [4]. In addition, the core genome contained the *blaF* gene (associated with β-lactam resistance) and the multidrug-resistance *mmr* genes.

There were 2956 accessory genes that were likely acquired or lost after the isolates diverged and may have helped each isolate adapt to its unique conditions. Compared to the core genes, a smaller proportion of the accessory genes could be assigned to a functional group (Fig. 2c). The genes in this group could contain errors that prevented identification or be specialized genes that have yet to be characterized.

### Chromosomal analysis of NJH_MKUB1 and NJH_MKUB2

The completeness of the NJH_MKUB1 and NJH_MKUB2 genomes allowed the chromosomes to be assessed independently from the plasmids. As an environmental organism, *M. kubicae* is equipped with chromosomal genes to help survival in excessive and limited nutrient conditions. For example, the chromosome contains genes that help tolerate the metals arsenate (*arsB*, *arsC*), copper (*copC*, *mmcO*) and zinc (*zitB*). Nutrient sequestering is important for surviving in nutrient-poor environments and *M. kubicae* has the tools to sequester copper (*ccs1*, *ccsA*), magnesium, cobalt and manganese (*corA*), zinc (*znuB*, *znuC*), and iron (*irtA*) (Fig. 1).

Iron scavenging is also an important mechanism used by *M. tuberculosis* to survive within human macrophages. When infected by mycobacteria, macrophages lower the levels of available iron in order to starve the bacteria of an essential nutrient [32]. To overcome this response, *M. tuberculosis* utilizes special membrane proteins encoded by *mmpL4/mmpS4* and *mmpL5/mmpS5* to secrete siderophores that scavenge much-needed iron. In turn, the siderophores are synthesized by genes *mbtA–N* [32]. The *M. kubicae* chromosome contains the *mmpL4/mmpS4* and *mmpL5/mmpS5* genes to produce the membrane proteins and some of the *mbt* genes needed to synthesize siderophores which may help the clinical isolates scavenge iron during macrophage infection.

The ESX type VII secretion system complex has also been linked to virulence and macrophage survival in mycobacteria. The *M. kubicae* genome contains some genes from each of the five different ESX systems, but only a complete copy of ESX-1. Though not found in *Mycobacterium abscessus *, ESX-1 is conserved in the pathogenic mycobacteria *M. tuberculosis *, *M. leprae* and *Mycobacterium marinum *, where it secretes various Esx, Esp and PE/PPE proteins to prevent phagosome maturation and disrupt host immunity [10, 33–35]. A closer phylogenetic relative of *M. kubicae *, *Mycobacterium smegmatis *, has an ESX-1 system, but unlike *M. tuberculosis *, ESX-1 in *M. smegmatis* is also involved in conjugal DNA transfer, suggesting that the exact function is context dependent [35].

*M. kubicae* contains both Esx genes (*esxA*, *esxB*), but only one of the Esp genes (*espB*). The few PE/PPE genes found in *M. kubicae* have been implicated in infection persistence (PE3) and iron sequestering (PE5) [36, 37]. Also present are the ESX system associated genes *mycP1–mycP5*. It is unclear whether the ESX-1 system and other ESX-related genes in *M. kubicae* assist organism survival in human macrophages like in *M. tuberculosis* or if these genes serve an unrelated mechanism such as in *M. smegmatis *.

Having these virulence genes available in the chromosome provides flexibility for opportunistic pathogens to infect a host when the conditions become favourable. It is also possible that these genes perform different primary functions within the context of *M. kubicae* compared to *M. tuberculosis *.

### Nine plasmids identified in *M. kubicae*

Many of the plasmids identified in NJH_MKUB1 and NJH_MKUB2 contribute some type of resistance, tolerance or biosynthesis genes that may be beneficial and could assist in the transition from environmental organisms to human pathogens. For example, the ESX-5 system has been associated with virulence in *M. marinum* [34] and with persistence in *M. tuberculosis* [35]. Plasmid pMKUB2_1 appeared to have delivered the ESX-5 system to NJH_MKUB2, possibly enhancing the ability of this strain to infect its human host.

The plasmid diversity observed among the *M. kubicae* strains gives the species another method of genomic flexibility. NJH_MKUB1, NJH_MKUB2 and JCM 13573 contained two, five and two plasmids, respectively, but only shared a few genes and regions. Plasmids pMKUB1_1 and pMKUB2_2 resembled regions in an unnamed plasmid in *M. paragordonae *, but lacked a number of genes that were already present in the *M. kubicae* chromosomes. As mobile genetic elements, the IS elements may have been involved in shuffling sequences between plasmids and into the *M. kubicae* chromosome. Harbouring the *M. paragordonae* version of the plasmid would have then been genetically redundant for these strains.

Many of the other plasmids contain resistance, tolerance or persistence genes that have a potential benefit to clinical *M. kubicae* isolates. The variation in plasmid constituency between these *M. kubicae* isolates indicates that the strains incorporated plasmids independently and maintained the plasmids that helped each strain in its specific conditions.

### Conclusion

The complete genomes of *M. kubicae* provide a scaffold to investigate the intraspecies genomic variation of NTM that are capable of causing human disease. Since *M. kubicae* infections are less common than other NTM species infections, the genetic characteristics, plasmid content and pathogenic potential have not been fully investigated to date. However, as this analysis of the *M. kubicae* genome demonstrates, virulence factors that may enhance pathogenic potential are present throughout the genome of this environmental micro-organism that can cause human disease. Genetic elements can be incorporated on the stable chromosome or transferred through mobile plasmids. This study emphasizes the importance of investigating the genomes of diverse species of NTM in order to further our understanding of genetic elements and genomic diversity that may influence the ability of environmental mycobacteria to become opportunistic pathogens. Specifically, the identification of potential virulence factors, drug-resistance genes and plasmids using complete genome information sheds light on this area. We have demonstrated that hybrid assembly using Illumina and ONT MinION reads is an approach that is well suited to address these needs in NTM.
